# Development and Application of Nasal Spine-Guided Classification for Maxillary Sinus Pneumatization

**DOI:** 10.3390/jcm15062124

**Published:** 2026-03-11

**Authors:** Ahmed M. Kabli, Rawan K. Kamal, Rayan M. Meer, Albraa Alolayan, Mohamed Omar Elboraey

**Affiliations:** 1 Department of Preventive Dental Sciences, College of Dentistry, Taibah University, Madinah 42353, Saudi Arabia; amkabli@taibahu.edu.sa (A.M.K.); rkamal@taibahu.edu.sa (R.K.K.); rmeer@taibahu.edu.sa (R.M.M.); 2 Department of Oral and Maxillofacial Diagnostic Sciences, College of Dentistry, Taibah University, Madinah 42353, Saudi Arabia; abolayan@taibahu.edu.sa; 3 Periodontology, Oral Diagnosis and Oral Radiology Department, Faculty of Dentistry, Tanta University, Tanta 31527, Egypt

**Keywords:** 3D, classification of maxillary sinus, pneumatization

## Abstract

**Background/Objectives**: The aim of this study was to develop a novel Nasal Spine-Guided Classification for assessing the alveolar vertical extension of the maxillary sinus and to evaluate its anatomical relationship with the roots of the posterior teeth using CBCT in a Saudi subpopulation. **Methods**: Maxillary sinus pneumatization was measured using cone-beam computed tomography for 380 patients. The assessment was performed along a horizontal plane extending between anterior and posterior nasal spine. In addition, pneumatization was evaluated in edentulous areas, and between the roots of multi-rooted teeth. Maxillary sinus membrane thickness was also measured. The results were expressed as mean, median and interquartile range, and considered statistically significant at a *p*-value < 0.05. **Results**: The mean maxillary sinus pneumatization on the left side was 8.8 ± 4.32 mm, and 8.58 ± 4.85 mm on the right side, with no statistically significant difference. The median of pneumatization in the edentulous area and between the roots on left side were 5.1 and 3.8 mm respectively, while on the right side, the median pneumatization was 5.03 and 3.04 mm. In addition, the proximity of the maxillary root apices to the sinus floor revealed a zero distance in 80.49% of the roots on the left side and in 79.48% on the right side. Furthermore, the results indicated no statistically significant association between maxillary sinus membrane thickness and pneumatization in the edentulous area. **Conclusions**: CBCT analysis revealed a predominance of advanced maxillary sinus pneumatization (Class III) and a high frequency of direct contact between posterior maxillary root apices and the sinus floor in the studied population. Additionally, no significant association was identified between maxillary sinus membrane thickness and sinus pneumatization in edentulous areas.

## 1. Introduction

The nose and paranasal sinuses constitute a unified functional complex and represent an essential component of the respiratory system. The maxillary sinus is the largest and the first developed paranasal sinus, and it is situated within the body of the maxilla. The alveolar process of the maxilla provides structural support for the maxillary teeth and constitutes the inferior boundary of the maxillary sinus. Moreover, the traditional concept of functionally dividing the maxillary sinus into an inferior dentoalveolar component managed by dental specialties and a superior component containing the sinus ostium under rhinologic care is no longer supported in contemporary anatomical and clinical understanding [1].

The maxillary sinus approximates a pyramidal configuration, with its base oriented toward the nasal cavity and its blunt apex directed toward the zygomatic bone. It contains several anatomical recesses, including the alveolar recess extending toward the roots of the maxillary teeth; the zygomatic recess extending toward the zygomatic bone; the palatine recess, a variable extension located between the floor of the nasal cavity and the palatal vault; and the infraorbital recess, which extends superiorly. The maxillary sinus is bounded by six walls: the superior, anterior, lateral, and medial walls, which are relatively broad, and the posterior and inferior walls, which are comparatively narrow [1].

The Schneiderian Membrane is the mucous membrane of the maxillary sinus that appears on radiographic examination as a thin and smooth soft tissue density. It is technically a mucoperiosteum, as the respiratory mucosa (consisting of pseudostratified ciliated columnar epithelium and a vascular lamina propria) is intimately adhered to the underlying periosteum. This periosteal component, which lines the inner bony walls of the sinus, contains osteogenic stem cells that play a vital role in bone graft healing during sinus floor elevation procedures [2].

Regarding the thickness of the mucous membrane of the maxillary sinus, there is a general agreement that exceeding 2 or 3 mm is considered pathological mucosal hypertrophy. Additionally, the mucous membrane thickness is greater in males than females. In both genders, the membrane thickness gradually decreases from the anterior to the posterior region [3]. There are numerous causes for mucosal thickening or hypertrophy, many of which are related to dental conditions, such as periapical infection, which is the most common, in addition to advanced periodontitis, oroantral communication, and recurrent or repeated surgical procedures related to the maxillary sinus. While odontogenic infections constitute a significant source, non-odontogenic conditions, particularly upper respiratory tract infections and allergic rhinitis, are among the most prevalent causes that must be taken into account during radiographic evaluation [1]. A retrospective study of a large study group using maxillofacial CT found that a healthy maxillary sinus with normal mucosal thickness is the least common at 40%, compared to the presence of diseases or thickening of the mucous membrane at 60% [4].

Epidemiological data indicate that maxillary sinus opacification, as well as unilateral inflammation of the anterior sinus group, is attributable to odontogenic causes in up to 75% of maxillary sinus cases and in approximately 25–40% of anterior sinus cases. Accordingly, a more comprehensive understanding of maxillary sinus pathophysiology, particularly the relationship between sinus infection and mucosal membrane thickening, and sinus pneumatization necessitates further detailed clinical and radiographic investigations [5].

Maxillary sinus septa are thin, shell-like bony lamellae that may present in linear or curved configurations or assume a morphology resembling an inverted Gothic arch. They most commonly originate from the inferior or inferolateral walls of the maxillary sinus; however, septa arising from the anterior wall have also been reported and constitute approximately 23% of all maxillary sinus septa. Septa located in the anterior region of the maxillary sinus are frequently associated with the presence of Haller’s cells. Using panoramic radiography to evaluate these septa reduces their actual detection rate; therefore, the most accurate and only precise method to evaluate these septa is computed tomography or cone-beam computed tomography [6,7].

Two principal types of maxillary sinus septa are recognized: developmental (primary) septa, which are present in both dentate and edentulous patients and typically appear as prominent, well-developed bony partitions; and acquired (secondary) septa, which develop following tooth loss as a result of sinus pneumatization and alveolar bone resorption. Primary and secondary septa have been reported in approximately 50% of posterior maxillary segments. Bony projections measuring ≤2.5 mm in height are preferably not classified as septa and are more appropriately described as bony ridges. The presence of maxillary sinus septa increases the technical complexity of maxillary sinus floor elevation procedures and has been associated with reduced thickness of the Schneiderian membrane [6,7].

A direct correlation exists between the overall size of the maxillary sinus and the vertical position (depth) of its sinus floor in alveolar process. In most individuals, the maxillary sinus floor is located approximately 3–5 mm inferior to the nasal floor [8,9]. Anatomical variations in the adult population indicate that the sinus floor lies below the level of the nasal floor in approximately 20% of cases, while it is positioned at the same level in nearly 15% of individuals [10].

Owing to the two-dimensional nature of panoramic radiography, an apparent spatial relationship is frequently observed between the apices of the maxillary posterior teeth and the floor of the maxillary sinus, often creating a false impression of root penetration into the sinus cavity [11]. Previous studies have reported that apparent root penetration into the maxillary sinus is observed in approximately 50% of cases when evaluated using panoramic radiographs; however, this percentage drops significantly to 27% when verified using Cross-sectional imaging (CBCT) [12].

Detailed studies using cone-beam computed tomography (CBCT) have categorized the relationship between the root apices and the sinus floor as follows, 35% of cases show an intimate relationship (contact) between the apex and the sinus floor, 25% of cases show the apex located exactly at the level of the sinus floor, 10% of cases demonstrate the apex appearing to be inside the sinus. Coronal CBCT sections reveal that, in such cases, the sinus floor frequently extends between adjacent roots rather than merely surrounding them, and 65% of the remaining cases show the sinus floor positioned above the root apices [13].

The average vertical distance between the maxillary sinus floor and the root apices varies according to tooth type, with the first premolar averaging approximately 7 mm and the second molar averaging about 2 mm. The shortest distance is most frequently observed at the distobuccal root apex of the second molar. Moreover, evidence suggests that the mean distance between the root apex and the sinus floor tends to decrease progressively with increasing patient age [13].

Based on an analysis of the existing literature and available data, it has been found that considerable variability exists in the size, shape, and extent of the maxillary sinus, along with the alveolar recess and their relationship to the roots of maxillary teeth, and sinus pneumatization and all its extensions along with their causes. However, no study has been found to cover all these aspects and explore possible correlations between all these different anatomical variations. This need has been felt more in the absence of any study done on this subject in Saudi Arabia. Hence, this study was undertaken to cover all these aspects.

## 2. Materials and Methods

Nearly 3974 cone-beam computed tomography (CBCT) scans archived at the College of Dentistry, Taibah University, Saudi Arabia, were retrospectively reviewed. Following screening and application of predefined inclusion and exclusion criteria, a total of 380 CBCT scans were included in the final analysis.

The inclusion criteria comprised adult patients aged ≥17 years, availability of high-quality CBCT scans without motion or metallic artifacts allowing adequate differentiation of anatomical structures, and complete visualization of bilaterally fully developed maxillary sinuses. All CBCT scans had been acquired for diagnostic or treatment planning purposes [8].

Scans were excluded if there was a history of facial or maxillary sinus trauma, previous sinus or sinonasal surgery, maxillary sinus elevation procedures, or sinus grafting. Additional exclusion criteria included the presence of cleft lip or palate, craniofacial anomalies or syndromes, severe image artifacts, incomplete clinical or demographic data, or incomplete visualization of the maxillary sinus [8].

The sample size was calculated a priori based on maxillary sinus pneumatization as the primary outcome measure. Prevalence of maxillary sinus pneumatization (pneumatization’ is defined as the extension of the sinus floor inferior to the level of the nasal floor) ranging from 40% to 50% depending on previous CBCT based studies. Assuming that an expected prevalence of 45%, a confidence level of 95%, and a margin of error of 5%, the standard formula for estimating proportions was applied (*n* = (*z*2 × *p* × (1 − *p*))/*d*2) as n represents the required sample size, *z* denotes the z-score corresponding to the selected confidence level (1.96), *p* represents the expected prevalence (0.45), and *d* indicates the margin of error (0.05). Based on the above parameters, the minimum sample size required was calculated to be 380 CBCT scans. This sample size is in accordance with previous studies on the anatomical variations and morphological characteristics of the maxillary sinus by means of CBCT [8].

Ethical clearance for this investigation was obtained from the Research Ethics Committee, College of Dentistry, Taibah University, Saudi Arabia (Approval No.: TUCDREC/280425/AKabli). This investigation has been undertaken in strict accordance with ethical research principles. All CBCT scans were treated with very high levels of confidentiality, and the information of the patients was anonymized before analysis. There were no instances of accessing or disclosing any kind of information related to the patients.

All CBCT scans were performed in accordance with a standardized imaging protocol designed by the College of Dentistry, Taibah University. Standardized protocol parameters during image acquisition were as the following 90 kV voltage, 4 mA current, 8.01 s exposure time, a dose area product coefficient of 684 mGy*cm^2^, and a voxel size of 160 μm. Using standardized imaging parameters in image acquisition was intended to ensure standardization of measurements and to reduce possible bias. Images were obtained using a Carestream cone-beam computed tomography device (Carestream Dental, Rochester, NY, USA). Images were then imported using Blue Sky Bio software version 4.

All radiographic evaluations were retrospectively conducted. Detection bias was minimized through anonymization and coding of radiographs, thereby ensuring blinding to patient identity and study group. All radiographic evaluations were conducted by two independent, calibrated examiners following a standardized evaluation protocol. Prior to extraction, a calibration session was conducted to determine evaluation criteria. Inter-rater reliability was also conducted on 20% of the sample, which was randomly selected.

### 2.1. Maxillary Sinus Pneumatization Using 3D CBCT Analysis

For the purpose of this clinical research study, maxillary sinus pneumatization has been operationally defined as the vertical extension of the maxillary sinus into the alveolar region. The extension was also quantified in terms of distance in millimeters.

The level of maxillary sinus pneumatization was assessed by taking advantage of the 3D view offered by cone-beam computed tomography (CBCT), whose results were analyzed by the BlueSky Bio software package (Blue Sky Plan 4, Blue Sky Bio, Libertyville, IL, USA), which offers the option of trimming the 3D view precisely. The axial plane was first created at the level of the horizontal reference from the anterior nasal spine to the posterior nasal spine, following the axial plane representing line viewed by the panoramic image, thus exposing the maxillary sinus on the axial view at the level of the two nasal spines on the 3D view. Two additional cuts on the sagittal planes were then created on the 3D view at the level of the floor of the maxillary sinus, following the deepest contour of the maxillary sinus on the right and left sides, respectively. The depth of the maxillary sinus was then measured from its axial cut (representing the plane from the anterior nasal spine to posterior nasal spine) to its deepest contour. The depth of the maxillary sinus formed the basis of a new classification system of maxillary sinus pneumatization, with the use of the anterior nasal spine as a reference point.

**Figure 1:**
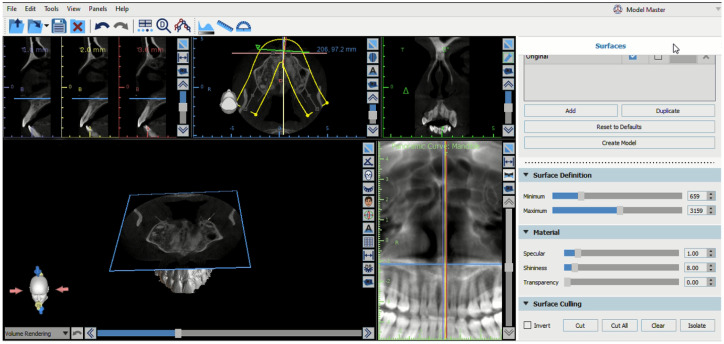
Axial 3D view in BlueSky Bio after sectioning at the level of the anterior nasal spine, aligned with the panoramic view to confirm correct positioning.

**Figure 2:**
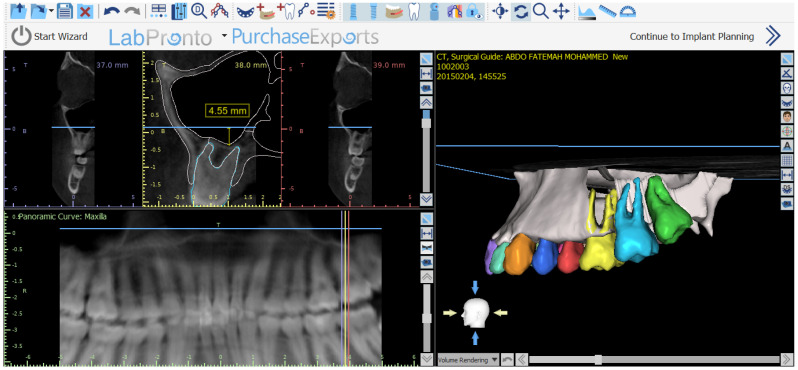
3D and panoramic views after positioning the axial plane at the level of the anterior nasal spine, showing measurement of maxillary sinus pneumatization on the panoramic view.

The classification consisted of three categories. In Class 1, the distance between the axial plane at the nasal spine and the deepest point of the maxillary sinus was from 0–4 mm. Class 2 represented distances between 5 and 8 mm, while Class 3 included distances exceeding 8 mm as shown in Figure 1 and Figure 2.

### 2.2. Sinus Pneumatization in Edentulous Areas

Maxillary sinus pneumatization in the edentulous area was evaluated on cone-beam CT scans using the ITK-SNAP software package (Version 4.4.0, U.S. National Institute of Biomedical Imaging and Bio Engineering). A horizontal reference line was first drawn connecting the apices of the adjacent teeth (mesial and distal to the extraction site). The distance from this reference line to the deepest point of the sinus pneumatization was then measured on the sagittal view. Measurement accuracy was subsequently verified at the same point using the coronal view.

### 2.3. Sinus Pneumatization in Multi-Rooted Teeth

**Figure 3:**
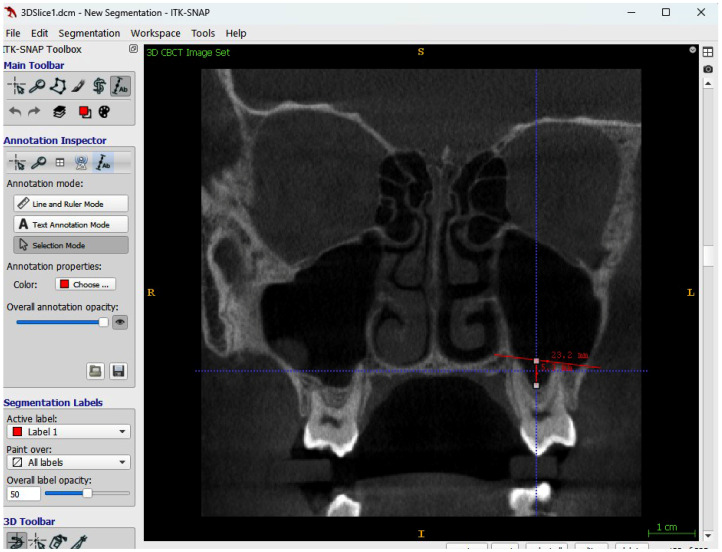
Cross sectional view from ITK-SNAP software showing measurement of sinus pneumatization in multi-rooted teeth.

Sinus pneumatization between the roots of maxillary multi-rooted teeth was assessed on CBCT using the cross-sectional view by ITK-SNAP software. A reference line was drawn connecting the apex of the highest buccal root to the apex of the palatal root and served as the baseline. From this line, the distance to the deepest point of sinus pneumatization between the roots was measured in millimeters to evaluate pneumatization within the root region (Figure 3).

### 2.4. Proximity of the Maxillary Root to the Sinus

The distance from the apices of the maxillary teeth located beneath the maxillary sinus to the inferior border of the sinus cortical bone was measured. Measurements were intentionally performed to exclude the thickness of the cortical bone. Specifically, the distance was recorded from the midpoint of each root apex following precise adjustment of the sagittal plane in CBCT images using ITK-SNAP software. Sagittal slices were individually optimized for each root to ensure accurate alignment with the long axis of the root apex and to obtain the most representative measurement.

### 2.5. Maxillary Sinus Membrane Thickness

**Figure 4:**
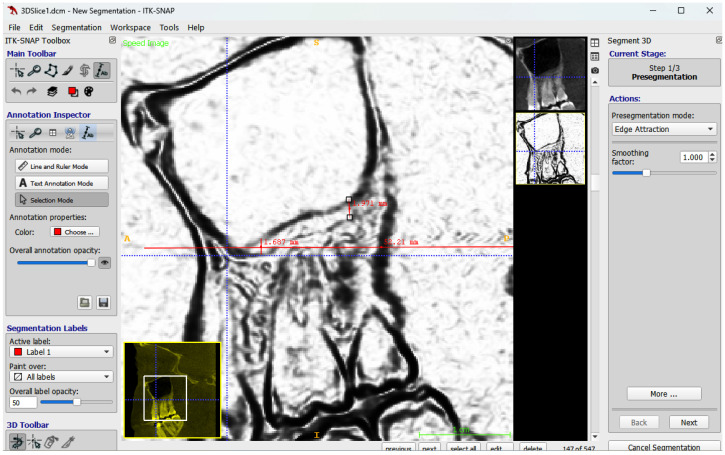
Sagittal view from ITK-SNAP software showing sinus membrane thickness measurement at two points.

In addition, the thickness of the maxillary sinus membrane was measured at two points in sagittal views. A sagittal section of the CBCT image is selected along the arch curve, which is aligned to the midline of the dental arch. The ITK-SNAP software is set to pre-segmentation mode, selecting the ‘edge attraction’ option to better visualize the sinus membrane. The deepest point of the maxillary sinus was identified, and the membrane thickness was measured at this location; the sinus membrane thickness was also measured at the highest point of the sinus floor (Figure 4).

## 3. Results

Statistical analysis was done by SPSS v29 (IBM Inc., Chicago, IL, USA). Shapiro-Wilks test and histograms were used to evaluate the normality of the distribution of data. Quantitative variables were presented as mean and standard deviation (SD) and compared between the two groups utilizing unpaired Student’s *t*-test. Quantitative non-parametric data were presented as median and interquartile range (IQR) and compared between the two groups utilizing the Mann–Whitney test and compared between the measurements utilizing Wilcoxon test. Qualitative variables were presented as frequency (%) and were analyzed utilizing the Chi-square test or Fisher’s exact test when appropriate. A two-tailed *p* value < 0.05 was considered statistically significant.

For measuring the reliability of the study, Cohen’s kappa was used for categorical data, and the Intraclass Correlation Coefficient (ICC) was used for continuous measurements with a two-way random effects model (single measurement, absolute agreement). Discrepancies were resolved by consensus. The results showed strong examiner agreement with high values of Cohen’s kappa ranging from 0.82 to 0.91 (95% CI: 0.75–0.96) and ICC ranging from 0.87 to 0.95 (95% CI: 0.81–0.98.

The age range of the participants varied from 17 to 61 years, with a mean age of 33.5 years and standard deviation of ±12.98 years. The sample had an equal number of males and females in terms of gender distribution, i.e., 50% male and 50% female. In total, 380 cases were used in the final analysis, as shown in Table 1.

### 3.1. Sinus Pneumatization

**Figure 5:**
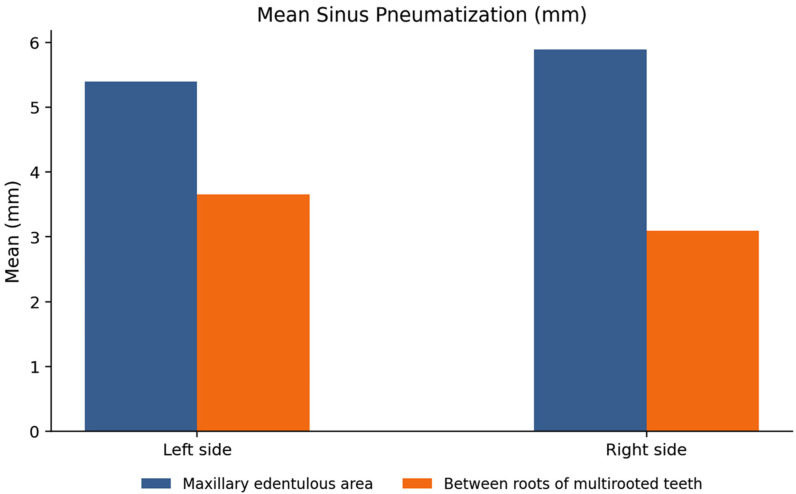
Mean of maxillary sinus pneumatization in mm at edentulous area and between roots for left and right side.

A comparative evaluation of the right and left maxillary sinus revealed gender variations in sinus pneumatization parameters. On the left side, the median (interquartile range) pneumatization value was 9.53 mm, and Class 1 was 13.42%, Class 2 was 30%, and Class 3 was 56.67%. The median value of pneumatization in the edentulous area was 5.10 mm, while between the roots of multi-rooted teeth was 3.8 mm as shown in Table 2. On the right side, the median (IQR) for sinus pneumatization classification values was 8.47 mm, with Class 1 representing 16.84%, Class 2 by 30%, and Class 3 by 56.15%. The median pneumatization was 5.03 mm in the edentulous area and 3.04 mm between the molar roots as shown in Table 3. Comparison between males and females showed no statistically significant differences in most measurements, except in the region of multi-rooted molar teeth in left side, where males demonstrated significantly greater sinus pneumatization than females (*p* = 0.022). No statistically significant differences were observed between the right and left sides (Figure 5).

### 3.2. Proximity of the Maxillary Root to the Sinus

On the left side, out of the 1040 teeth were examined, 837 teeth, representing 80.49%, had a distance of zero millimeters between the root apex and the lower cortex of the maxillary sinus. Meanwhile, 203 teeth, accounting for 19.51%, exhibited a measurable distance greater than zero. These distances ranged from 2.0 mm to 13.8 mm. The mean distance was 6.02 mm, with a standard deviation of ±3.5 mm, reflecting the anatomical variability on the left side of the maxilla as shown in Table 2.

On the right side, out of 902 teeth, 717 teeth (79.48%) had a zero-millimeter distance between the root apex and the inferior cortical plate of the maxillary sinus, while 185 teeth (20.52%) had a distance greater than zero, ranging from 1.3 mm to 12.5 mm. The mean distance was 5.46 mm, with a standard deviation of ±3.0 mm as shown in Table 3.

**Figure 6:**
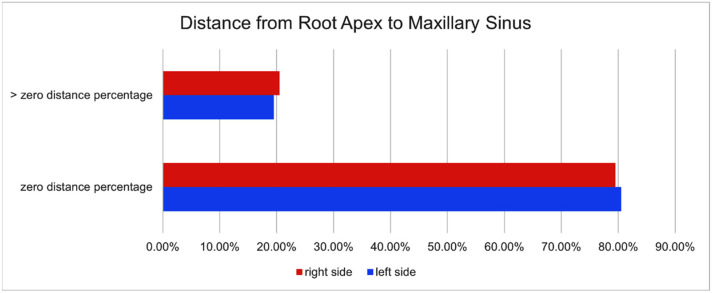
The percentage of teeth with a distance of zero millimeters between the root apex and the lower cortex of the maxillary sinus.

In the gender-based analysis, it was observed that 78.59% of the teeth showed zero distance in males, whereas 21.00% showed a distance greater than zero. Similarly, in females, it was observed that 81.28% teeth showed zero distance, whereas 18.18% showed a distance greater than zero. The *p*-value for comparison between males and females was 0.762, showing no significant difference between the two genders (Figure 6).

### 3.3. Membrane Thickness

A notable difference was observed in maxillary sinus membrane thickness on the left side between the nearest and farthest sites. The membrane thickness at the nearest site was significantly greater than at the farthest site (*p* = 0.023), with median values of 2.00 mm and 1.475 mm, respectively. On the right side, no statistically significant difference was detected between the nearest and farthest sites, where the median membrane thickness measured 1.59 mm and 1.57 mm, respectively as shown in Table 4

Furthermore, a correlation analysis demonstrated no statistically significant association between maxillary sinus membrane thickness at the nearest site and sinus pneumatization in the edentulous area on either side as shown in Table 5.

## 4. Discussion

This study was conducted on 380 cone-beam computed tomography (CBCT) scans obtained from a Saudi population to evaluate and analyze various anatomical parameters of the maxillary sinus. All CBCT examinations were retrieved from the College of Dentistry, Taibah University. Both the right and left maxillary sinuses were assessed, and the study sample comprised an equal distribution of male and female participants (50% each).

Maxillary sinus pneumatization was measured using a horizontal line (from the anterior nasal spine to posterior nasal spine) down to the deepest point in the maxillary sinus. On the left side the median was 9.53 mm, while on the right side it was 8.47 mm, with no statistically significant differences between males and females. Compared to previous studies that indicated that the average using the same reference line ranges between 3 and 5 mm at different age group, the current results confirm a significant pneumatization in the maxillary sinuses of the Saudi population in Madinah [8,9].

In the study by Lovasova et al., entitled “3D CAD/CAM Imaging of the Maxillary Sinus in the Edentulous Process,” adult male and female human cadaver heads were examined, with ages ranging from 37 to 83 years. A three-dimensional CAD/CAM software was used to scan the solid impression of the maxillary sinus and determine its true morphology. The pneumatization distance was measured using the same reference horizontal line, extending from the anterior nasal spine to the deepest point of the maxillary sinus, and their reported mean ranged between 1 and 1.5 cm [10]. In comparison with the current study, the pneumatization distance reached 12.83 mm. This indicates a reasonable agreement between the two studies, even with that Lovasova et al.’s investigation was conducted on edentulous participants explaining the greater values for their study [10].

In this study, a new classification system was introduced to assess maxillary sinus pneumatization using horizontal reference line drawn through the anterior nasal spine and posterior nasal spine as a reference plane. This classification was primarily performed on three-dimensional segmentations of the maxillary sinuses to clearly visualize all anatomical details and accurately identify the true deepest point of sinus positioning. For additional precision, measurements were taken by using vertical line from the horizontal reference line to the deepest point of the maxillary sinus, specifically to its inner wall, in order to minimize the effects of varying bony thickness in the region. The classification system had three levels: Category I ranged from 0 to 4 mm, Category II ranged from 5 to 8 mm, and Category III exceeded 8 mm. Most of the measurements fell under Category III, which accounted for 56.57% on the left side and 53.15% on the right side.

The Nasal Spine-Guided Classification system that is proposed in the current context has the distinct advantage of utilizing the anterior nasal spine, a bony structure that is radiopaque, as the reference point for the classification. The classification system has the advantage of providing a highly standardized and reproducible method of assessing the relationship between the sinus floor and the posterior roots, as opposed to the conventional systems that may rely on the anatomy of the soft tissues in the region, which may not be as well-defined. The classification system can thus be utilized to effectively predict the risk of oro-antral communication, thus providing a better surgical strategy in the extraction of the posterior maxillary teeth or in procedures of sinus augmentation.

In another study conducted by Wu et al. (2022), [8] CBCT was used to evaluate the degree of pneumatization of the maxillary sinus in the alveolar process of individuals of different age groups. The evaluation of pneumatization was done based on the method described by Cavalcanti and Wagner. This method required the determination of a horizontal line that connected the anterior and posterior nasal spine (nasal floor). The location of the sinus floor was then classified as positive and negative. A positive value indicated that the sinus floor was below the nasal floor, while a negative value indicated that the sinus floor was above the floor of the nose.

Using the same approach, the vertical distance between this horizontal reference line (nasal floor) and the deepest point of sinus pneumatization was measured. The mean pneumatization values reported by Wei et al. ranged between 3.77 mm and 4.07 mm across the different age groups. In comparison, the results of the present study demonstrated substantially greater values, indicating a notably higher degree of sinus pneumatization among the Saudi population.

Numerous classifications have been proposed to evaluate the vertical pneumatization of the maxillary sinus alveolar recess within the alveolar bone. One example is the classification proposed by Niu et al. [14] which aimed to define the geometrical pattern of sinus pneumatization Another system is the Kalavagunta & Reddy [15] which categorized the maximum horizontal and vertical dimensions of the maxillary sinus were measured and compared with the corresponding dimensions of the orbit in the same coronal section. (mild, moderate, and severe), using CT scans. In contrast, our classification effectively measures the distance of sinus pneumatization in millimeters, providing an accurate and precise vertical assessment of maxillary sinus pneumatization.

With regard to our classification of vertical pneumatization of the maxillary sinus alveolar recess, horizontal reference line was drawn through the anterior nasal spine and posterior nasal spine. This approach was first introduced by Cavalcanti et al. [16] defines a horizontal plane that extends from the anterior to the posterior nasal spine and is referred to as the palatine plate. The classification of the positions relative to the plane is as follows: positions above the plane are interpreted as having a positive correlation, positions on the same plane are interpreted as “at level,” and positions below the plane have a negative correlation, which corresponds to true alveolar pneumatization.

In the present study, it was proposed that the process of pneumatization of the sinus can be categorized into three distinct classes, depending on the distance between the floor of the sinus and the deepest point of pneumatization. These categories have been defined as Category 1, where the distance ranges from 0 to 4 mm, Category 2, where the distance ranges from 5 to 8 mm, and Category 3, where the distance extends beyond 8 mm. This proposed system of categorization has been designed to align with the widely accepted Alveolar Ridge Classification system proposed by Carl Misch, which categorizes the available bone height. This would ensure a better correlation between the proposed system of categorization and the established scientific principles. For example, the lowest category of pneumatization, which extends from 0 to 4 mm, would correlate with the highest bone availability category proposed by Carl Misch, SA1. Similarly, the other categories, which range from 5 to 8 mm and beyond 8 mm, would correlate with the intermediate and lowest bone availability categories, SA2 and SA3, proposed by Carl Misch [17].

The methodology used to measure the variables in this study utilized a sophisticated capability offered by the BlueSky Bio software that facilitates three-dimensional interpretation and slicing of 3D View CBCT scans. This enables simultaneous sagittal slicing of the 3D View for both the right and left maxillary sinuses within a single viewing interface. In addition, axial slicing can also be used to virtually remove the roof of the maxillary sinus and access the palatine plane within the same 3D View. This facilitates increased accessibility and convenience of measurement while at the same time ensuring increased accuracy by eliminating the need to continually realign sagittal and coronal slices to identify the palatine plane. In addition, this enables simultaneous measurement while validating the alignment and symmetry of the palatine plane through both panoramic and sagittal views.

However, another set of studies, done by Sharan and Madjar [18] as well as by Pei et al. [19], had adopted the AS as a standard, with some examination of whether this particular sinus was pneumatized or not. These researchers have divided sinus pneumatization into two varieties, namely normal as well as pneumatized or extended varieties. The sinus would be described as normal if there was a particular distance between the sinus floor and apices of the posterior teeth of the maxilla, and pneumatized when the sinus floor was contiguous with the apices of these teeth.

On the other hand, Cavalcanti et al. [16] determined the maxillary sinus pneumatization level by finding the difference between two values. The first value was the distance from the floor of the nasal cavity to the deepest point of maxillary sinus pneumatization, whereas the second value was the distance from the floor of the nasal cavity to the apex of the existing dentition. This approach is similar to the approach used in the current study, where the distance from the horizontal reference line drawn through the apices of the teeth adjacent to the edentulous region to the deepest point of maxillary sinus pneumatization was determined. According to Cavalcanti et al., the average was approximately 2.93 mm, with a standard deviation of 0.9 mm. The median values in the current study were 5.1 mm on the left side and 5.03 mm on the right side. The results obtained from the current study and Cavalcanti et al.’s study indicate a divergence in the results, suggesting that the Saudi population has greater maxillary sinus pneumatization in the edentulous region compared to other populations [16].

In an independent study, Shadia et al., [20] carried out a retrospective study in the city of Madinah to assess the maxillary sinus pneumatization in female patients using digital panoramic radiographs. The researchers divided the sinus pneumatization of the maxilla into three categories, of which the first represents the actual pneumatization. The researchers followed the same protocol as the current study to measure the distance from the root apex to the deepest point of the sinus pneumatization. The researchers reported the mean values ranging between 2.82 mm and 3.22 mm. On the other hand, the results of the current study showed considerable variation in the values, with the mean being 5.4 mm on the left and 5.98 mm on the right. In addition, the prevalence of sinus pneumatization ranged between 36.7% on the left and 35% on the right. This difference could be due to the fact that the current study employed cone-beam computed tomography, which offers three-dimensional imaging and hence an accurate analysis. The current study included male and female participants in equal proportions, i.e., 50% of the total participants [20].

As for the extension of the maxillary sinus between the roots of multi-rooted maxillary teeth, this measurement was quantified by creating a horizontal reference line on the cross-sections of teeth that showed extension of the maxillary sinus. The horizontal reference line connected the apex of the palatal root with the longest buccal root. The measurement was taken from this reference line to the deepest maxillary sinus pneumatization between the roots. When compared with the study conducted by Ji et al. in 2025, [21] which investigated the influence of maxillary sinus pneumatization anatomical patterns in relation to tooth roots for endodontic microsurgery planning, their classification system divided interradicular sinus extension into five types. Types III, IV, and V were comparable to the patterns observed in the present study, with reported mean extension values ranging between 1.4 mm and 1.5 mm. In contrast, the present study demonstrated greater interradicular sinus pneumatization, with mean values of 3.09 ± 1.45 mm on the right side and 3.66 ± 1.40 mm on the left side [21].

The prevalence of interradicular sinus pneumatization was found to be 43.3% for the left side and 50% for the right side. The pattern of sinus pneumatization among the teeth that exhibited this feature revealed that the maxillary first molar tooth (tooth 16) accounted for 50% of all cases on the right side and 33% of all cases on the left side. When the sample population was stratified by gender, all cases of sinus pneumatization among females were maxillary first molars for both the right and left sides. Similarly, among males, the maxillary first molar tooth accounted for 80% of all cases on the right side and 57% of all cases on the left side. In summary, all these findings point to the fact that sinus pneumatization between the roots tends to localize to the maxillary first molar region.

The sagittal CBCT measurements showed that the distance from the midpoint of the maxillary tooth root apices to the inferior cortical boundary of the maxillary sinus was zero in 80.49% of the cases on the left side, while on the right side, the result was 79.48%. There was no indication of root penetration into the sinus cavity. Although the sinus pneumatization was observed to extend between the root apices, there was no indication of root intrusion into the sinus. This is in agreement with the concept of the maxillary sinus being a “balloon-like” structure that completely surrounds the root apices without any anatomical interference. This has significant clinical implications, which could result in the increased prevalence of oroantral communication in the extraction of teeth, as seen in the population of Al-Madinah Al-Munawwarah [7,20].

Although the study emphasizes the role of sinus pneumatization and root proximity in the formation of Osteoantral Communication (OAC), it is important to note that Osteoantral Communication is a multifactorial complication, and apart from the above factors, surgical factors, such as the application of excessive force during extraction or improper use of dental elevators, are often responsible for Osteoantral Communication. In addition, pathological conditions, such as chronic periapical periodontitis, may cause resorption of the thin layer of cortical bone between the root and the maxillary sinus, thereby greatly increasing the likelihood of Osteoantral Communication, irrespective of the initial root proximity. Moreover, the presence of a complex root form, such as a divergent or dilacerated root, may require more aggressive surgical procedures, thereby increasing the likelihood of Osteoantral Communication [22].

The results of the study show that the median thickness of the maxillary sinus membrane in the region of sinus pneumatization was 2.0 mm on the left and 1.95 mm on the right. Conversely, the median thickness of the maxillary sinus membrane in the region without sinus pneumatization was 1.47 mm on the left and 1.75 mm on the right. A statistically significant difference was found in the left region, indicating a noticeable increase in the thickness of the sinus membrane during sinus pneumatization. When compared with the results of a previous study in the southwestern region of Saudi Arabia, the mean thickness of the maxillary sinus membrane in the present study was found to be higher than the reported mean of 1.17 to 1.46 mm [23].

Based on the aforementioned findings, the results have revealed a marked increase in the expansion of maxillary sinus pneumatization from the nasal floor, including the pneumatization of edentulous areas and interradicular spaces, and the prominence of the maxillary root apices towards the sinus without actual perforation. This is due to the presence of a zero distance between the root apex and the floor of the sinus. Hence, the maxillary sinus pneumatization could be viewed as a possible primary cause for the increased rate of occurrence of oro-antral communication in the extraction of the maxillary root.

Our evaluation of maxillary sinus pneumatization, performed in edentulous areas and in the interdental spaces between multi-rooted teeth, offers a precise classification that can be easily related to a clinical context. By providing a precise evaluation of the proximity between the roots of posterior teeth and the sinus floor, this classification is expected to improve the planning of implant procedures. Thus, even if the main purpose of this study has been to provide a better understanding of the anatomical variations, it has shown how easily cone-beam computed tomography can be incorporated into daily clinical practice, enhancing risk assessment and surgical precision.

Limitations: The study also failed to examine the particular anatomical variations that could influence maxillary sinus pneumatization, including the presence of maxillary sinus septa and their influence on maxillary sinus pneumatization, as well as the volumetric dimensions of the maxillary sinus. Variations in sinus floor morphology may still differ across larger, multi-ethnic populations. Future multi-center studies are recommended to validate these findings across a broader demographic spectrum.

Future recommendations: It is recommended that future investigations incorporate three-dimensional (3D) printing of the maxillary sinus derived from segmented 3D imaging data. This approach would facilitate a precise and comprehensive evaluation of anatomical variations, including the morphology and orientation of sinus septa, as well as accurate quantification of sinus volumetric dimensions. Future multi-center studies are recommended to validate these findings across a broader demographic spectrum.

## Figures and Tables

**Table 1:** Demographic data of the participants studied.

Variables		(n = 380)
Age (years)	Mean ± SD	33.5 ± 12.98
	Range	17–61
Sex	Male	190 (50%)
	Female	190 (50%)

Data are presented as mean ± SD or frequency (%).

**Table 2:** Maxillary sinus pneumatization of the studied patients on the left side.

Variables		(n = 380)
Sinus pneumatization classification (mm)	Median	9.53
	IQR	5.6–12.17
	Class I	51 (13.42%)
	Class II	114 (30%)
	Class III	215 (56.57%)
Sinus pneumatization edentulous area (mm)	Median	5.1
	IQR	4.35–6.5
Sinus pneumatization between roots of multi-rooted teeth (mm)	Median	3.8
	IQR	2.4–4.8

Data is presented as mean ± SD, median (IQR) and as frequency (%).

**Table 3:** Maxillary sinus pneumatization of the studied patients on the right side.

Variables		(n = 380)
Sinus pneumatization classification (mm)	Median	8.47
	IQR	5.75–12.83
	Class I	64 (16.84%)
	Class II	114 (30%)
	Class III	202 (53.15%)
Sinus pneumatization edentulous area (mm)	Median	5.03
	IQR	2.78–6.73
Sinus pneumatization between root of multi-rooted teeth (mm)	Median	3.04
	IQR	2.43–4.15

Data is presented as mean ± SD, median (IQR) and as frequency (%).

**Table 4:** Comparison between membrane thickness at nearest and at the farthest on the left and the right sides of the studied patients.

		Membrane Thickness at Nearest (n = 380)	Membrane Thickness at Farthest (n = 380)	*p* Value
At the left side	Median	2	1.475	0.023
	IQR	1.33–2.6	1.13–2.58	
At the right side	Median	1.95	1.75	0.073
	IQR	1.53–2.68	1.23–2.1	

Data is presented as median (IQR).

**Table 5:** Correlation between sinus pneumatization in edentulous area and membrane thickness at nearest on both sides of the studied groups.

		Sinus Pneumatization Edentulous	
		Left side	Right side
Membrane thickness at nearest	r	0.563	−3.68
	*p* value	0.07	0.295

r: Correlation coefficient.

## Data Availability

The data presented in this study are available on request from the corresponding author.

## Abbreviations

The following abbreviations are used in this manuscript:

CBCT	Cone-Beam Computed Tomography
SD	Standard Deviation
ICC	Intraclass Correlation Coefficient
